# Hydrogen-bond potential for ice VIII-X phase transition

**DOI:** 10.1038/srep37161

**Published:** 2016-11-14

**Authors:** Xi Zhang, Shun Chen, Jichen Li

**Affiliations:** 1 Institute of Nanosurface Science and Engineering & Guangdong Provincial Key Laboratory of Micro/Nano Optomechatronics Engineering, Shenzhen University, Guangdong, 518060, China; 2 School of Physics and Astronomy, the University of Manchester, Manchester, M13 9PL, UK

## Abstract

Repulsive force between the O-H bonding electrons and the O:H nonbonding pair within hydrogen bond (O-H:O) is an often overlooked interaction which dictates the extraordinary recoverability and sensitivity of water and ice. Here, we present a potential model for this hidden force opposing ice compression of ice VIII-X phase transition based on the density functional theory (DFT) and neutron scattering observations. We consider the H-O bond covalent force, the O:H nonbond dispersion force, and the hidden force to approach equilibrium under compression. Due to the charge polarization within the O:H-O bond, the curvatures of the H-O bond and the O:H nonbond potentials show opposite sign before transition, resulting in the asymmetric relaxation of H-O and O:H (O:H contraction and H-O elongation) and the H^+^ proton centralization towards phase X. When cross the VIII-X phase boundary, both H-O and O:H contract slightly. The potential model reproduces the VIII-X phase transition as observed in experiment. Development of the potential model may provide a choice for further calculations of water anomalies.

The physical origin and theoretical reproduction of the measured anomalies of water and ice remains a great challenge^1^,^2^,^3^,^4^,^5^,^6^,^7^,^8^,^9^. For example, under compression of the O:H-O bond, the H-O covalent bond becomes longer while the total O–O distance is shortened, leading to the proton centring in the O–O of ice VIII-X phase transition^2^,^10^,^11^,^12^,^13^,^14^. The behaviour of the proton is so strange that thermal fluctuation^15^ and quantum effect of nuclei^16^ were both considered contributing to the ambiguous behaviour. Unlike other materials whose phonons were harden by external pressure, ice-VIII demonstrated the anomalous softening of the H-O vibration mode at frequency greater than 3000 cm^−1^ while stiffening of the O:H vibration mode at frequency lower than 400 cm^−1^^17^. Ice melts under compression and freezes again when the pressure is relieved, evidencing extraordinary recoverability of O:H-O bond^21^. Hydrogen bond interaction potential, typical double-well potentials of the symmetrical^22^ and the asymmetrical^23^ forms, is still under debate.

An accurate description of the H-bonding between water-water molecules is widely recognised as the crucial factor in the understanding of water anomalies^9^,^24^,^25^,^26^. Progresses has been made by considering the flexibility of the covalent bonding in water/ice in models such as the flexible-SPC^27^ and TIP4PF^28^. Kumagai *et al*. have proposed a flexible KKY potential which has three separate terms: *V*_*HO*_*, V*_*HH*_ and *V*_*OO*_ in order to account the intra- and inter-molecular interactions separately^11^ and adopted by our previous MD calculation^29^. However, since those models have not distinguished O–O linked by O:H-O or not, the reproduction of the proton centring of ice VIII-X has seldom been reported. *Ab initio* MD (AIMD) enabled people to use the *ab initio*-derived force field, though time-expensive. Bernasconi *et al*.^15^,^30^ reproduced the ice VIII/VII/X phase transition by AIMD-derived lattice relaxation and infrared spectra. Sugimura *et al*.^9^,^11^ determined the intermediate structure during the ice VII/X phase transition using AIMD. Katoh *et al*.^31^,^32^ confirmed the fast protonic diffusion coefficient at high temperature molecular phase of ice VII. However, previous researches mostly focused on the structure determination or proton transferring, while the mechanism of the proton centring and the cooperative relaxation of the O:H and H-O are not fully understood. A potential model for time-saving calculations of the proton centring in the ice VIII/X phase transition is still lacking.

Recent progresses find that the hidden force^33^, i.e. the repulsive force between the H-O bonding electrons and the O:H nonbonding pair within a O:H-O bond, should play a significant role in various anomalies of water and ice, such as extraordinary recoverability, skin lubricity, etc.^33^,^34^,^35^,^36^. For example, in the temperature range from 250 K to 277 K, H-O covalent bond exhibit the normal thermal expansion, while O:H nonbond shrinks due to the reducing repulsive force, leading to the contraction of the total O–O distance^34^,^36^. At the skin of ice, due to the decrease of the molecular coordination, H-O covalence bond contracts spontaneously to lower the cohesive energy, and hence O:H nonbond is polarized, leading to the surface lubricity^35^,^37^. This force is beyond the conventional intra- and inter-molecular interactions but depending on the existence of the O:H-O link, i.e. if there is no O:H-O bond like between two O atoms in difference sublattice in ice VIII, hidden force (3 times stronger than Van de Vaals force) interaction would not show^38^. However, it has still seldom reported to address the hidden force of O:H-O bond in a H-bond model, although the force plays a significant role in altering the properties of water and ice.

In this paper, in order to investigate the strange behaviour of proton centring in phase VIII-X transition, we clarified a hidden force model considering the quantum interactions between electron clouds of covalent bond and nonbond and reproduced the anomalous behaviour of proton in ice VIII-X transition.

## Potential Model Proposed

**Figure 1:**
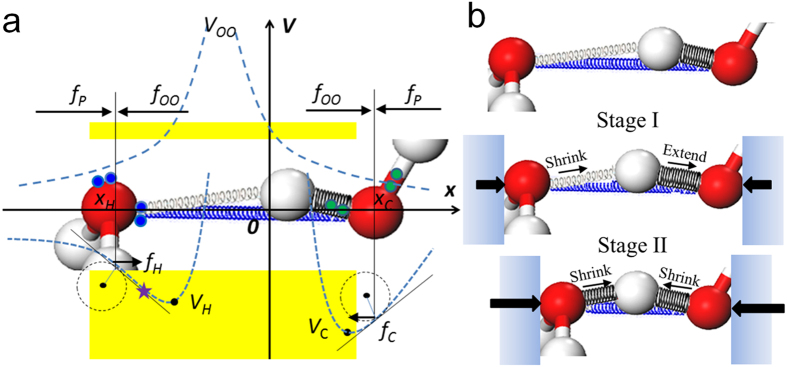
(**a**) Hidden force model for O: H-O bond. x_H_ and x_C_ are the O atomic position counting from x = 0 (H atom). *f*_*OO*_ is the hidden repulsive force. *f*_*P*_ is the external compression force. *f*_*H*_ and *f*_*C*_ are the recovery force of O:H and O-H, in value of slope of the potential curves (blue dash lines) of *V*_*H*_ and *V*_*C*_. Star represents the curvature turning point. (**b**) Two stages of O:H-O relaxation under compression. Stage I (ice VIII): A small external force compresses the O:H at first since O:H interaction is weaker than O-H. *f*_*oo*_ is then increased to extend O-H. Stage II (ice X): under a large external force, *f*_*H*_ goes across the curvature turning point, leading to the shrinks of both O:H and O-H.

Based on our extensive investigation of water anomalies^21^,^34^,^35^,^36^, we proposed hidden force model for dynamics of the “O: H-O” bond under external pressures, as shown in Fig. 1a. In the model, “O: H-O” bond is similar to be connected by three “non-harmonic converted to anharmonic springs”. Black spring represents covalent bonding of H-O (denoted as subscript C); Grey spring represents van der Waals interaction between lone pair (blue circles) and proton of O:H (denoted as subscript H); Blue spring represents repulsive interaction between bonding electron pair and lone pair (denoted as subscript OO, but the interaction is not the conventional O–O coulomb repulsion). A water molecule is surrounded by four nearest neighbours (Pauling’s ice rule). Four identical O: H-O forms a tetrahedron as the basic unit of water structure (Supplementary Fig. S1). The bond length (*x*), forces (*f*) and potential(*V*) of O:H, H-O and O–O need to reach to a balance under external force (*f*_*p*_). Figure 1b shows two stages of O:H-O relaxation under compression.

In stage I, a small external force compresses the O:H at first since O:H interaction is weaker than O-H. The repulsive *f*_oo_ then is increased by the contraction of O…O, leading to H-O extension. In stage II, under a large *f*_*p*_, *f*_*H*_goes across the curvature turning point, leading to the shrinks of both O:H and O-H. Intermediate phases exist during the transition where proton disordered symmetrization occurs with delocalized proton in a relatively broad potential well. The delocalization of proton (or proton-transfer) may attributed to quantum effect of nuclei^16^,^39^, or to thermal fluctuations obtained by AIMD^15^. In this work, we focus on the proton centring due to electron interactions within the O:H-O bond.

Without external force, taking centre proton as reference, three springs can reach to equilibrium with their forces along the directions as indicated in Fig. 1a. Due to the *f*_oo_, both O atoms are pushed away a little from their lowest-energy positions in the potentials of isolated bonds. Thus, the initial potential recovery forces *f*_*C*_ and *f*_*H*_ will both point inward to center proton; while repulsive force *f*_OO_ will point outward. Supposing 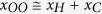, at equilibrium, total forces (force values) added on O atoms should both be zero:


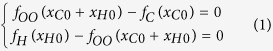


where, *x*_*C0*_ and *x*_*H0*_ (length values in positive numbers) are the equilibrium position of O atoms counting form *x* = 0. Thus, in equilibrium without external force,


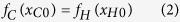


Considering an identical force *f*_*p*_ at both sides, the forces satisfy:


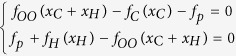


if


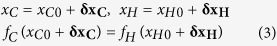


Supposing the displacement 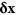 is small enough to consider *k* as a constant, since 
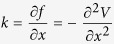
, combining eq. (2) and eq. (3), we can get:


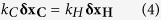


Eq. (4) tells us:

i) The values of 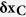 and 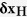 should be determined by the potential forms of V_H_ and V_C_. Since O:H bond is much longer and weaker than H-O bond, V_H_ is more flat (smaller curvature) than V_C_, i.e. |k_C_| >| k_H_|. Thus, the value of 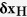 is always larger than 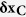.

ii)If 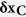 and 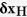 have opposite signs, i.e. *x*_*C*_ and *x*_*H*_ do not both increase or decrease together, *k*_*H*_ and *k*_*C*_ must have opposite sign with each other, which means the curvatures of potentials are opposite, as shown in Fig. 1a.

iii) If 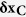 and 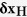 have same signs, i.e. *x*_*C*_ and *x*_*H*_ both increase or decrease together, *k*_*H*_ and *k*_*C*_ must have the same sign.

Due to the charge polarization within the O:H-O bond, the curvature of the H-O potential has the opposite sign with O:H potential in the low-pressure region, leading to the contraction of O:H bond but extension of H-O bond and the proton centring. Hence, for O: H-O bond relaxation under external *f*_*p*_, there are two stages as shown in Fig. 1b:

i) Stage I (ice VIII): in quasi-static process, external force contracts the O:H bond but extends the H-O bond with the total O–O length shortened, when *k*_*C*_ ∙ *k*_*H*_ < 0. Both the work of *f*_*P*_ and the change of *V*_*C*_ provide energy for increase of *V*_*H*_ and work of f_OO_:
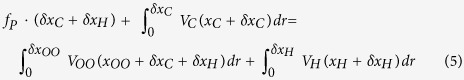


ii) Stage II (ice X): external force is strong enough to make *x*_*H*_ shrink over the curvature turning point (star in Fig. 1) and *k*_*C*_ ∙ *k*_*H*_ > 0. External force contract both segments, pushing O atoms back towards the energy-lowest positions, compressing all the three springs. In this case, *f*_*P*_ and energy increases for both segments provides the sharp increase of the *V*_*OO*_:


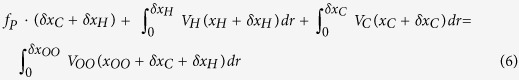


### Calculations and Verifications

To verify and quantify our potential model, we performed density functional theory (DFT) calculation using CASTEP. The generalized gradient approximation (GGA) function of PW91, HCTH and RPBE were used to describe the exchange–correlation effects (results show little differences among the different functions and hence the PW91 results were presented here). The van der Waals force was also examed by adopting the DFT-D, its effect to the stability of the ice structures has reported, a small effect of red-shifts (~2 meV) for the main peaks of the vibrational spectra was observed. The phonon spectra were calculated using the CASTEP module with finite displacement method. The force constants produced by the CASTEP for the phonon spectra calculations were obtained from the output files. The force for the atom *i*, *f*_*i*_ (= d*E*/d*r*_*i*_), is simply the derivative of the total energy *E*. By applying a second small displacement for the atom *j*, the force constant *k*_ij_ (= d*U*/d*r*_*i*_d*r*_*j*_) is obtained for the pair atoms *i* and *j*. A force constant matrix **k** for the unit cell is a 3 *N*×3 *N* matrix (where *N* is total number of atoms in the unit cell) and were constructed based on above procedure.

**Figure 2:**
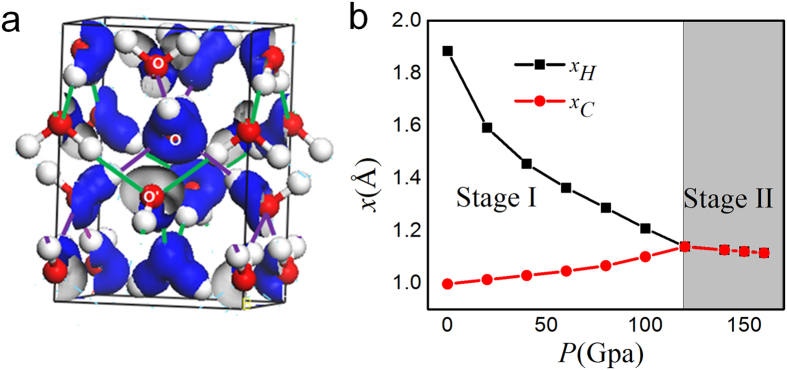
(**a**) Structure of ice VIII. Two nested sublattices connected by green and purple lines respectively are shown. The oxygen atoms in the same sublattice were denoted as O and in the other sublattice as O’. Deformation electron density was plotted as blue isosurface. (**b**) DFT-derived bond length of O:H(*x*_*H*_) and O-H(*x*_*C*_) versus pressure. The changing trend of *x*_*H*_ and *x*_*C*_ falls into two stages.

Ice VIII has a cubic structure containing two sets of interpenetrated ice sub-lattices as shown in Fig. 2a. Hence, each water molecule has 8 nearest neighbours, 4 of them are H-bonded and the others are not, at almost the same O–O distance. The oxygen atoms in the same sublattice were denoted as O and in the other sublattice as O’. Blue isosurface of deformation electron density (density difference before and after bonding,) indicates the position of the bonding electron pair and the O:H nonbond pair. The charge distribution in a water molecule was polarized in the tetrahedral directions by the O:H-O bond. Figure 2b shows the pressure-dependent bond length of O:H(*x*_*H*_) and O-H(*x*_*C*_). Proton centring is obtained as pressure increases, in accord with the experimental observation^2^,^12^ and other DFT calculation^33^. The changing trends of *x*_*H*_ and *x*_*C*_ verify the two stages of O:H-O under compression. *x*_*H*_ shrinks and *x*_*C*_ extends in stage I. As pressure increases, *x*_*H*_ and *x*_*C*_ both shrink in stage II like normal material.

**Figure 3:**
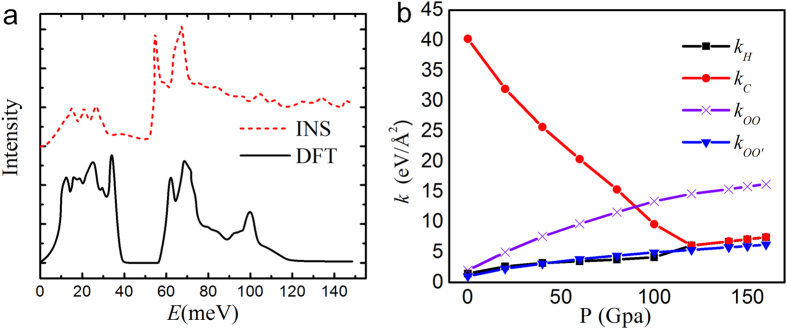
(**a**) Phonon spectra of ice VIII measured using inelastic neutron scattering and calculated uing DFT. (**b**) Force constants of O-H, O:H, O–O in O:H-O bonding and O–O’ without bonding.

The DFT calculation can reproduce phonon spectrum for Ice VIII as shown in Fig. 3a. The simulation reproduces the main features of inelastic neutron scattering (INS) spectrum^24^, such as the three peaks in the translational region (<50 meV) and the two sharp peaks of the librational region at about 65 meV. The small peak in the right hand side of librational region at about 100 meV was not shown in the INS spectra due to large Debye-Waller effect in the measured spectra which smeared the spectra dramatically at higher energy transfer. Force constants *k*_*C*_*, k*_*H*_ and *k*_*OO*_ were also obtained from the calculation of phonon spectrum of the ice VIII and are plotted as a function of pressure in Fig. 3b. Apart from the trend for the *k*_*C*_*, k*_*B*_ to merge at *x*_*OO*_ = 2.3 Å the force constants between the H-bonded O–O atoms *k*_*OO*_ also shows a rapid increase under the external pressure. For the non H-bonded O…O’ atoms between the two sub-lattices of ice VIII, a new force constant, *k*_*OO’*,_ was obtained which remains at small values around *k*_*H*_. This implies that the O–O interaction only becomes relevant when the two O atoms are linked by an H atom and hence this large value of *k*_*OO*_ is not due to the simple coulomb interaction between O atoms. As shown by the isosurface in Fig. 2a, charge distribution was polarized by O:H-O bonding, i.e. the electron clouds were accumulated in the line of O:H-O, leading to the increase of the repulsive force between bonding pair and nonbonding pair of O–O and the decrease of the interaction between O and O’.

### Potential Model Parametrization

**Figure 4:**
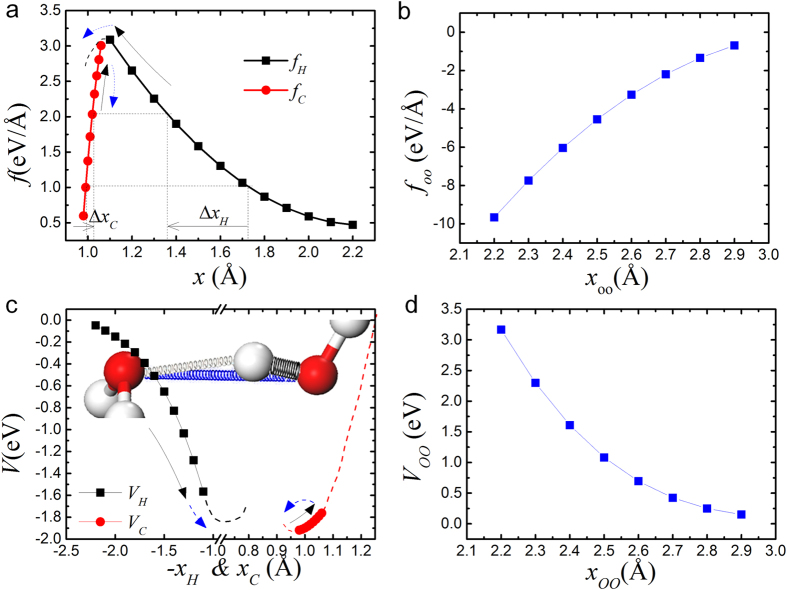
The DFT-fitting curves of bond length *x*-dependent (**a**), *f*_*C*_ and *f*_*H*_, (**b**), *f*_*OO*_, (**c**), *V*_*C*_ and *V*_*H*_, and (**d**), *V*_*OO*_ according to water model. Black arrows indicate the opposite relaxation in length of *x*_*C*_ and *x*_*H*_ in stage I; Blue arrows indicate the both decreasing in length of *x*_*C*_ and *x*_*H*_ in stage II.

Fitting from DFT-derived *k*-*x* results of stage I of P-dependent IceVIII (Fig. 3b), we get the relations among *k*_*i*_ and *x*_*i*_ (I = H, C, OO) according to our potential model,


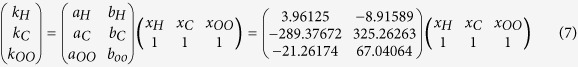


where we add negative sign on k_H_ since DFT only take the positive square root value. Based on *k*-*x* curve, we fit the expression of *f-x* and *V-x* curves (Supplementary Eq. S1 and S2). The shapes of *f-x* and *V-x* are plotted in Fig. 4.

In stage I, as *f*_*p*_ increases, *f*_*H*_ = *f*_*C*_ should also increases according to Eq.(3). Thus, *x*_*H*_ decreases while *x*_*C*_ increases as indicated by black arrows in Fig. 4a. In stage II, when *x*_*H*_ decreases to about 1.1 and reach to the curvature turning point, *f*_*H*_ changes to decrease and *x*_*C*_ will decreases back (as shown by blue arrows) to make *f*_*C*_ = *f*_*H*_. The absolute value of repulsive force *f*_*OO*_ always increases as distance *x*_*OO*_ decreses. We focus on the parametrization of stage I in this work since it is more interesting than the normal compression-contraction in stage II.

**Figure 5:**
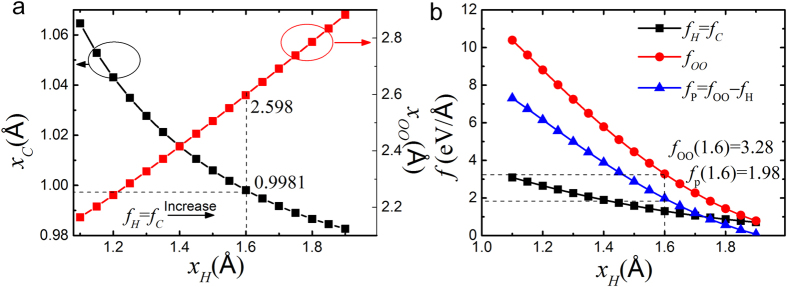
(**a**) Correlation between *x*_*H*_ and *x*_*C*_. (**b**) Correlation of *x*_*H*_, *f*_*P*_. *f*_*H*_, and *f*_OO_. Under external *f*_*p*_, the length and force relaxations inside the O:H-O bonding are all determined.

Hence, for a given *f*_P_, there is only one set of solutions of *x*_*H*_ and *x*_*C*_ obtained in Fig. 5. Figure 5a shows that, for a given *x*_*H*_, *f*_*H*_ = *f*_*C*_ gets the *x*_*C*_. Then, Fig. 5b shows that, *f*_*OO*_ can be calculated by *x*_H_ + *x*_*C*_ and *f*_P_ is correlated with *x*_H_ by *f*_*p*_ = *f*_*OO*_−*f*_*H*_. Hence, we have obtained the *f*_*P*_-*x*_*H*_ curve in Fig. 5b, from which a given external *f*_P_ can determine the relaxation of *x*_*H*_ and *x*_*C*_. Thus, the correlations among external compression force, the length relaxation of H-O and O:H bond, the forces of O-H, O:H and O:O are all determined. Figure 5b also indicates that, as *f*_P_ increases, *f*_OO_ rises much more significantly than *f*_*H*_. Thus, *f*_OO_ is indeed a hidden force that should not be ignored.

**Figure 6:**
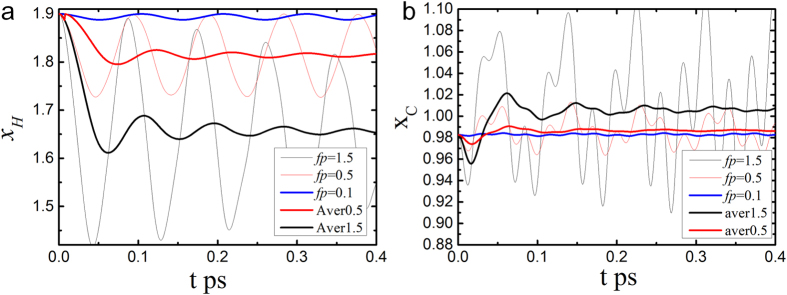
Time-dependent relaxation dynamics of O:H-O bond with the same initial lengths and different external forces. (**a**) *x*_H_ relaxes with time and (**b**), *x*_C_ relaxes with time under *fp* = 0.1, 0.5 and 1.5 eV/Å. The initial lengths are 1.9 and 0.98269 at 0 external force, which give initial energies for bonds to oscillate around the equbrillium point under pressures. *x*_H_ and *x*_C_ under *fp* = 0.5 and 1.5 eV/Å were taken average.

Based on our potential model, *foo*, *f*_*H*_ and *fp* are functions of *x*_*H*_ and *x*_*C*_ determined by Supplementary Eq. S1 and Fig. 4. We run the time-dependent dynamic process of external pressure on O:H-O bond in Fig. 6. The dynamic equation can be expressed as:


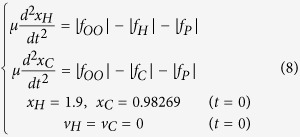


The initial condition of *x*_*H*_ and *x*_*C*_ are the lengths (Å) without pressure. The partial differential equation is solved by 4^th^ order Runge-Kutta method (Supplementary Method).

Figure 6 shows the *x*_*H*_ and *x*_*C*_ relaxation with the same initial lenghs and different external forces under *f*_*p*_ = 0.1, 0.5 and 1.5 eV/Å. The initial lengths are bond lengths at fp = 0, which give energies to atoms to oscillate. If the process is slow, i.e. initial coordinates are around the stable point, the oscillation will decrease. *x*_H_ and *x*_C_ under *fp* = 0.5 and 1.5 eV/Å were taken average as 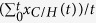. The results show that, as *fp* increases, *x*_*H*_ decreases from 1.890 to 1.811 and to 1.645 Å in average, while *x*_*C*_ increases from 0.983 to 0.992 and to 1.015 Å in average, agreeing well with proton centring obtained both in DFT calculation and experimental observation^2^,^12^.

**Figure 7:**
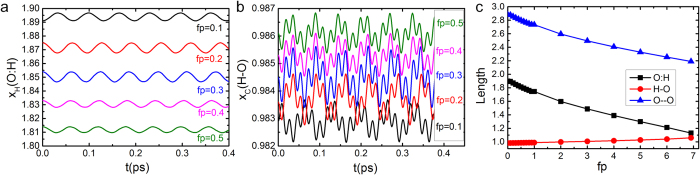
Time-dependent dynamics of O:H-O bond in the quasi-static process, with different initial bond lengths. (**a**) *x*_*H*_ relaxes with time and (**b**), *x*_*C*_ relaxes with time under *fp* from 0.1 to 0.5 eV/Å. The initial lengths at each *fp* were taken as the average bond lengths of the previous *fp*. Hence, the oscillations of bonds were largely reduced. (**c**), the average bond lengths derived at *fp* from 0.1 to 6.91 eV/Å. Proton centring occurs near *fp* = 6.91 eV/Å.

Figure 7 a and b show the time-dependent dynamics of O:H-O bond in the quasi-static process under *fp* from 0.1 to 0.5 eV/Å. The initial lengths at each *fp* were taken as the average bond lengths of the previous *fp*. Hence, bond length relaxes more stably with time. Figure 7c shows the average bond lengths obtained from the quasi-static states at different *fp*. The dynamic curves oscillated stably raise the reliability of the average lengths. Results show that as *fp* increases, O:H bond length decreases and H-O bond length increases, approaching to equal at about *fp*  = 6.91 eV/Å. the Therefore, upon compression, in the equilibrium status of O:H-O, proton and oxygen shift both towards the other oxygen while proton shift a little to the weaker O:H part. Besides, O:H vibrates much slower than H-O bond due to its weaker force constant, as indicated by the frequency of the relaxation curves.

The potential model of O:H-O can be further applied to water molecules and crystal structures considering the interactions of H—H and O–O’. V_OO’_ follows the weak dispersion interaction as indicated by the force constant developed by DFT in Fig. 3b, since electron cloud distributes mostly on the line of O:H-O bond as shown in Fig. 2a. Current potential model provides an efficient tool to investigate bond length relaxation, time-dependent proton dynamics, mass density and intermediate structure determination of dense ice phases.

## Conclusion

DFT and neutron scattering observations have enabled a O:H-O bond potential for the ice VIII-X phase transition under compression. Before transition, H-O undergoes elongation and the O:H compression because of the O:H-O bond segmental disparity and O-O repulsion; after transition, both H-O and O:H undergoes slight contraction because of the excessive compression. The effectiveness of current potential model was illustrated when dealing with H-bonded systems under pressure. More importantly, this additional force could provide the necessary mechanism to explain a range of water anomalies, for instance the complex phase diagram, i.e. its morphism, this is probably because the hydrogen bonds become very easy to buckle under the extra strong O–O interaction if the O:H-O is not in straight line. This hidden force, *f*_*OO*_ has often been overlooked experimentally in the past, perhaps because it is easily concealed by the *f*_*H*_ and *f*_*C*_. The hidden force potential model reproduced the contraction of O:H bond and extension of H-O bond and the proton centralization under compression. The potential model to develop further can serve as a key for theoretical reproduction of the O:H-O bond asymmetric relaxation under external stimuli.

## Additional Information

**How to cite this article**: Zhang, X. *et al*. Hydrogen-bond potential for ice VIII-X phase transition. *Sci. Rep*. **6**, 37161; doi: 10.1038/srep37161 (2016).

**Publisher’s note**: Springer Nature remains neutral with regard to jurisdictional claims in published maps and institutional affiliations.

## Supplementary Material

Supplementary Information

## References

[b1] Goncharov A. F., Struzhkin V. V., Mao H.-k. & Hemley R. J. Raman Spectroscopy of Dense H2O and the Transition to Symmetric Hydrogen Bonds. Phys. Rev. Lett. 83, 1998 (1999).

[b2] Benoit M., Marx D. & Parrinello M. Tunnelling and zero-point motion in high-pressure ice. Nature 392, 258–261 (1998).

[b3] Frenken J. W. M. & Oosterkamp T. H. MICROSCOPY When mica and water meet. Nature 464, 38–39, doi: 10.1038/464038a (2010). 20203595

[b4] Headrick J. M. . Spectral signatures of hydrated proton vibrations in water clusters. Science 308, 1765–1769, doi: 10.1126/science.1113094 (2005). 15961665

[b5] Gregory J. K., Clary D. C., Liu K., Brown M. G. & Saykally R. J. The Water Dipole Moment in Water Clusters. Science 275, 814–817, doi: 10.1126/science.275.5301.814 (1997). 9012344

[b6] Bjerrum N. Structure and properties of ice. Science 115, 385–390 (1952). 17741864 10.1126/science.115.2989.385

[b7] Soper A. K., Teixeira J. & Head-Gordon T. Is ambient water inhomogeneous on the nanometer-length scale? Proc. Natl. Acad. Sci. USA. 107, E44–E44, doi: 10.1073/pnas.0912158107 (2010). 20220097 PMC2851810

[b8] Zha C.-S., Hemley R. J., Gramsch S. A., Mao H.-k. & Bassett W. A. Optical study of H2O ice to 120GPa: Dielectric function, molecular polarizability, and equation of state J. Chem. Phys. 126, 074506, doi: 10.1063/1.2463773 (2007). 17328619

[b9] Iitaka T., Fukui H., Li Z., Hiraoka N. & Irifune T. Pressure-induced dissociation of water molecules in ice VII. Sci Rep 5, 12551, doi: 10.1038/srep12551 (2015). 26212425 PMC4515761

[b10] Loubeyre P., LeToullec R., Wolanin E., Hanfland M. & Husermann D. Modulated phases and proton centring in ice observed by X-ray diffraction up to 170 GPa. Nature 397, 503–506 (1999).

[b11] Sugimura E. . Compression of H2O ice to 126 GPa and implications for hydrogen-bond symmetrization: Synchrotron x-ray diffraction measurements and density-functional calculations. Physical Review B 77, 214103 (2008).

[b12] Kang D., Dai J., Hou Y. & Yuan J. Structure and vibrational spectra of small water clusters from first principles simulations. J. Chem. Phys. 133, 014302 (2010). 20614964 10.1063/1.3462278

[b13] Holzapfel W. B. On the Symmetry of the Hydrogen Bonds in Ice VII. The Journal of Chemical Physics 56, 712–715 (1972).

[b14] Bhatt H. . Hydrogen Bond Symmetrization in Glycinium Oxalate under Pressure. The Journal of Physical Chemistry B 120, 851–859, 10.1021/acs.jpcb.5b11507 (2016). 26730739

[b15] Bernasconi M., Silvestrelli P. L. & Parrinello M. Ab Initio Infrared Absorption Study of the Hydrogen-Bond Symmetrization in Ice. Phys. Rev. Lett. 81, 1235–1238 (1998).

[b16] Meng X. . Direct visualization of concerted proton tunnelling in a water nanocluster. Nat Phys 11, 235–239, doi: 10.1038/nphys3225 (2015).

[b17] Yoshimura Y., Stewart S. T., Somayazulu M., Mao H. & Hemley R. J. High-pressure x-ray diffraction and Raman spectroscopy of ice VIII. J. Chem. Phys. 124, 024502, doi: 10.1063/1.2140277 (2006). 16422606

[b18] Pruzan P. . Phase diagram of ice in the VII–VIII–X domain. Vibrational and structural data for stronglycompressed ice VIII. J. Raman Spectrosc. 34, 591–610 (2003).

[b19] Song M., Yamawaki H., Fujihisa H., Sakashita M. & Aoki K. Infrared absorption study of Fermi resonance and hydrogen-bond symmetrization of ice up to 141 GPa. Physical Review B 60, 12644 (1999). 10.1103/physrevb.54.15673 9985631

[b20] Santra B. . Hydrogen Bonds and van der Waals Forces in Ice at Ambient and High Pressures. Phys. Rev. Lett. 107, 185701 (2011). 22107644 10.1103/PhysRevLett.107.185701

[b21] Zhang X. . Ice Regelation: Hydrogen-bond extraordinary recoverability and water quasisolid-phase-boundary dispersivity. Scientific Reports 5, 13655, doi: 10.1038/srep13655 (2015). 26351109 PMC4563362

[b22] Teixeira J. High-pressure physics - The double identity of ice X. Nature 392, 232–233 (1998).

[b23] Kumagai T. Direct observation and control of hydrogen-bond dynamics using low-temperature scanning tunneling microscopy. Prog. Surf. Sci. 90, 239–291, doi: 10.1016/j.progsurf.2015.04.001 (2015).

[b24] Li J. & Ross D. K. Evidence for two kinds of hydrogen bond in ice. Nature 365, 327–329 (1993).

[b25] Zhang L.-J., Wang J., Luo Y., Fang H.-P. & Hu J. A novel water layer structure inside nanobubbles at room temperature Nuclear Science and Techniques 25, 060503, doi: 10.13538/j.1001-8042/nst.25.060503 (2014).

[b26] Gaffney K. J., Ji M., Odelius M., Park S. & Sun Z. H-bond switching and ligand exchange dynamics in aqueous ionic solution. Chem. Phys. Lett. 504, 1–6, doi: 10.1016/j.cplett.2011.01.063 (2011).

[b27] Toukan K. & Rahman A. Molecular-dynamics study of atomic motions in water. Physical Review B 31, 2643–2648 (1985). 10.1103/physrevb.31.2643 9936106

[b28] Lawrence C. P. & Skinner J. L. Flexible TIP4P model for molecular dynamics simulation of liquid water. Chem. Phys. Lett. 372, 842–847, doi: 10.1016/S0009-2614(03)00526-8 (2003).

[b29] Burnham C. J., Li J. C. & Leslie M. Molecular Dynamics Calculations for Ice Ih. The Journal of Physical Chemistry B 101, 6192–6195, doi: 10.1021/jp9632596 (1997).

[b30] Benoit M., Romero A. H. & Marx D. Reassigning Hydrogen-Bond Centering in Dense Ice. Phys. Rev. Lett. 89, 145501 (2002). 12366053 10.1103/PhysRevLett.89.145501

[b31] Cavazzoni C. . Superionic and Metallic States of Water and Ammonia at Giant Planet Conditions. Science 283, 44–46, doi: 10.1126/science.283.5398.44 (1999). 9872734

[b32] Eriko Katoh H. Y., Fujihisa H., Sakashita M. & Aoki K.. Protonic Diffusion in High-Pressure Ice VII. Science 295, 1264–1266, doi: 10.1126/science.1067746 (2002). 11847334

[b33] Sun C. Q., Zhang X. & Zheng W. The hidden force opposing ice compression. Chemical Science 3, 1455–1460 (2012).

[b34] Huang Y. . Hydrogen-bond relaxation dynamics: Resolving mysteries of water ice. Coord. Chem. Rev. 285, 109–165, doi: 10.1016/j.ccr.2014.10.003 (2015).

[b35] Zhang X. . Water Nanodroplet Thermodynamics: Quasi-Solid Phase-Boundary Dispersivity. The Journal of Physical Chemistry B 119, 5265–5269, 10.1021/acs.jpcb.5b00773 (2015). 25719395

[b36] Zhang X. . Water’s phase diagram: From the notion of thermodynamics to hydrogen-bond cooperativity. Prog. Solid State Chem. 43, 71–81, doi: 10.1016/j.progsolidstchem.2015.03.001 (2015).

[b37] Zhang X., Huang Y., Ma Z., Niu L. & Sun C. Q. From ice superlubricity to quantum friction: Electronic repulsivity and phononic elasticity. Friction 3, 294–319, 10.1007/s40544-015-0097-z (2015).

[b38] Shun C., Zhiping X. & Jichen L. The observation of oxygen—oxygen interactions in ice. New Journal of Physics 18, 023052, (2016).

[b39] Herrero C. P. & Ramírez R. Path-integral simulation of ice VII: Pressure and temperature effects. Chem. Phys. 461, 125–136, doi: 10.1016/j.chemphys.2015.09.011 (2015).

