# Supplementary material for: Hydrogen-bond potential for ice VIII-X phase transition
Source: Sci Rep. 2016 Nov 14;6:37161. doi: 10.1038/srep37161 (PMC5107924; doi:10.1038/srep37161)
Supplement: Supplementary Information [file srep37161-s1.pdf]

Supplementary Information:

Hydrogen-bond potential for ice VIII-X phase transition

Xi Zhang,[1*] Shun Chen,[2] Jichen Li[2*]

[1]*Institute of Nanosurface Science and Engineering, Shenzhen University, Guangdong, 518060, China*

[2]*School of Physics and Astronomy, the University of Manchester, Manchester, M13 9PL, UK*

1) O:H-O water structure:

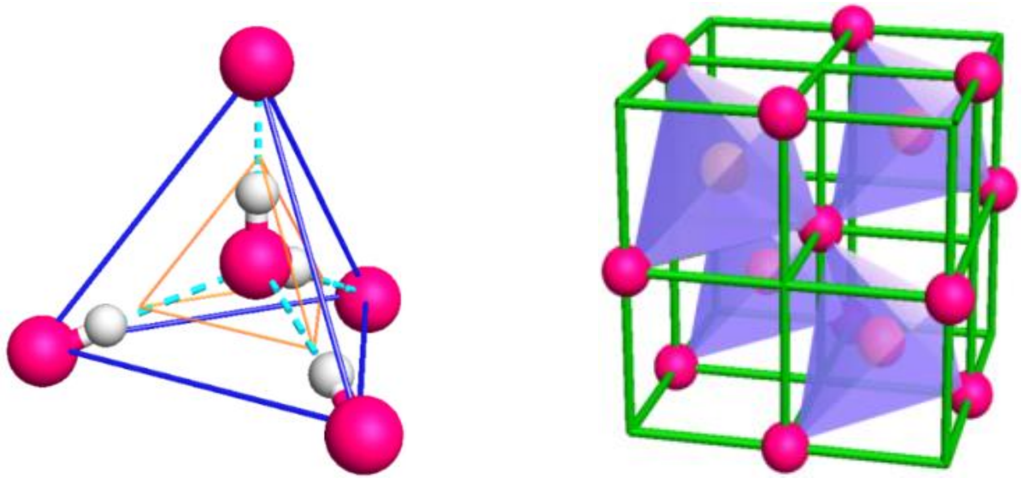

Figure S1 (a) An ideal tetrahedron that contains two equivalent H_2O molecules connected by four identical O:H-O bonds of different orientations. (b) Packing of the basic building block forms a diamond structure, which ensures the tetrahedral coordination of the central oxygen atom in the coordination origin.

2) Water model parameterization:

Fitting from DFT of P-dependent IceVIII of stage I, approximating $x_{OO} \cong x_H + x_C$, *k*-*x* curves are fitted in the paper.

$$\begin{pmatrix} k_H \\ k_C \\ k_{OO} \end{pmatrix} = \begin{pmatrix} a_H & b_H \\ a_C & b_C \\ a_{OO} & b_{oo} \end{pmatrix} \begin{pmatrix} x_H & x_C & x_{OO} \\ 1 & 1 & 1 \end{pmatrix} = \begin{pmatrix} 3.96125 & -8.91589 \\ -289.37672 & 325.26263 \\ -21.26174 & 67.04064 \end{pmatrix} \begin{pmatrix} x_H & x_C & x_{OO} \\ 1 & 1 & 1 \end{pmatrix}$$

Since $k_i = \frac{\partial f_i}{\partial x_i} = \frac{\partial^2 V_i}{\partial x_i^{\,2}} (i = H, C, OO)$, *f*-x and *v*-x are fitted as:

$$\begin{pmatrix} f_H \\ f_C \\ f_{OO} \end{pmatrix} = \begin{pmatrix} a_H/2 & b_H & 10.5 \\ a_C/2 & b_C & -179.2 \\ a_{OO}/2 & b_{oo} & -105.7 \end{pmatrix} \begin{pmatrix} x_H^2 & x_C^2 & x_{OO}^2 \\ x_H & x_C & x_{OO} \\ 1 & 1 & 1 \end{pmatrix}$$

(S1)

$$\begin{pmatrix} V_H \\ V_C \\ V_{OO} \end{pmatrix} = \begin{pmatrix} a_H/6 & b_H/2 & 10.5 & -8.6 \\ a_C/6 & b_C/2 & -179.2 & 62.9 \\ a_{OO}/6 & b_{oo}/2 & -105.7 & 111.2 \end{pmatrix} \begin{pmatrix} x_H^3 & x_C^3 & x_{OO}^3 \\ x_H^2 & x_C^2 & x_{OO}^2 \\ x_H & x_C & x_{OO} \\ 1 & 1 & 1 \end{pmatrix}$$

(S2)

3) O:H-O bond relaxation dynamics under compression:

Dynamic equation is expressed as:

$$\begin{cases} \mu \dfrac{d^2 x_H}{dt^2} = |f_{OO}| - |f_H| - |f_P| \\ \mu \dfrac{d^2 x_C}{dt^2} = |f_{OO}| - |f_C| - |f_P| \\ x_H = 1.9, x_C = 0.98269 \qquad (t=0) \\ v_H = v_C = 0 \qquad (t=0) \end{cases}$$

(S3)

μ is the effective mass, 1.16eV·ps^2/ Å^2

Substituting f_{OO}, f_H, f_C in the first and second equations with expressions in Eq. S1 and setting fp as 0.1eV/ Å for example, we can obtain the functions $S_H(x_H, x_C)$ and $S_C(x_H, x_C)$ in the right side of the equations. The partial differential equation is solved by 4th order Runge-Kutta method.

The second order partial differential equation is reduced into first order:

$$\begin{cases} \dfrac{dx_H}{dt} = v_H \\ \dfrac{dv_H}{dt} = S_H(x_H, x_C) \\ \dfrac{dx_c}{dt} = v_C \\ \dfrac{dv_C}{dt} = S_C(x_H, x_C) \end{cases}$$

(S4)

v_H and v_C are the velocities. Set vector **y** as the combination of the unknowns and **S** as the set of functions, the equation can be expressed as:

$y=(x_H, v_H, x_C, v_C)$, $y_0=(1.9,0,0.98269,0)$

$dy/dt=S(y,t)$

(S5)

Using 4th order Runge-Kutta method, the y(t) in eq.S5 can be solved as:

$f_1=dt*S(y(t))$

$f_2=dt*S(y(t)+f_1/2)$

$f_3=dt*S(y(t)+f_2/2)$

$f_4=dt*S(y(t)+f_3)$

$y(t+dt)=y(t)+(f_1+2*(f_2+f_3)+f_4)/6$

(S6)

The time interval d*t* is set small enough (0.1fs in this case) to make sure the precision. According to Eq S6, y(t+dt) can be obtained by the previous step y(t).
